# Sulfated β-glucan from *Agaricus subrufescens* inhibits flavivirus infection and nonstructural protein 1-mediated pathogenesis

**DOI:** 10.1016/j.antiviral.2022.105330

**Published:** 2022-05-06

**Authors:** Francielle Tramontini Gomes de Sousa, Scott B. Biering, Trishna S. Patel, Sophie F. Blanc, Carla M. Camelini, Dalila Venzke, Ricardo J. Nunes, Camila M. Romano, P. Robert Beatty, Ester C. Sabino, Eva Harris

**Affiliations:** a Division of Infectious Diseases and Vaccinology, School of Public Health, University of California, Berkeley, Berkeley, CA, 94720-3370, USA; b Departamento de Microbiologia, Imunologia e Parasitologia, Universidade Federal de Santa Catarina, Florianópolis, SC, 88.040-900, Brazil; c Departamento de Química, Universidade Federal de Santa Catarina, Florianópolis, SC, 88.040-900, Brazil; d Instituto de Medicina Tropical, Faculdade de Medicina da Universidade de São Paulo, São Paulo, SP, 05403000, Brazil; e Laboratório de Virologia (LIMHC 52), Hospital das Clínicas da Faculdade de Medicina da Universidade de São Paulo (HCFMUSP), São Paulo, SP, Brazil

**Keywords:** Sulfated polysaccharides, Vascular leak, Nonstructural protein 1, Dengue virus, Zika virus, Flavivirus

## Abstract

Despite substantial morbidity and mortality, no therapeutic agents exist for treatment of dengue or Zika, and the currently available dengue vaccine is only recommended for dengue virus (DENV)-immune individuals. Thus, development of therapeutic and/or preventive drugs is urgently needed. DENV and Zika virus (ZIKV) nonstructural protein 1 (NS1) can directly trigger endothelial barrier dysfunction and induce inflammatory responses, contributing to vascular leak *in vivo.* Here we evaluated the efficacy of the (1–6,1–3)-β-D-glucan isolated from *Agaricus subrufescens* fruiting bodies (FR) and its sulfated derivative (FR-S) against DENV-2 and ZIKV infection and NS1-mediated pathogenesis. FR-S, but not FR, significantly inhibited DENV-2 and ZIKV replication in human monocytic cells (EC_50_ = 36.5 and 188.7 μg/mL, respectively) when added simultaneously with viral infection. No inhibitory effect was observed when FR or FR-S were added post-infection, suggesting inhibition of viral entry as a mechanism of action. In an *in vitro* model of endothelial permeability using human pulmonary microvascular endothelial cells (HPMECs), FR and FR-S (0.12 μg/mL) inhibited DENV-2 NS1- and ZIKV NS1-induced hyperpermeability by 50% and 100%, respectively, as measured by Trans-Endothelial Electrical Resistance. Treatment with 0.25 μg/mL of FR and FR-S inhibited DENV-2 NS1 binding to HPMECs. Further, FR-S significantly reduced intradermal hyperpermeability induced by DENV-2 NS1 in C57BL/6 mice and protected against DENV-induced morbidity and mortality in a murine model of dengue vascular leak syndrome. Thus, we demonstrate efficacy of FR-S against DENV and ZIKV infection and NS1-induced endothelial permeability *in vitro* and *in vivo*. These findings encourage further exploration of FR-S and other glycan candidates for flavivirus treatment alone or in combination with compounds with different mechanisms of action.

## 1. Introduction

Dengue and Zika viruses, members of the *Flaviviridae* family, genus *Flavivirus*, are mosquito-borne viruses that cause major medical and public health problems worldwide. Dengue virus (DENV) causes an estimated 100 million infections, 51 million dengue cases, and up to 4 million hospitalizations annually worldwide (Cattarino et al., 2020). During 2013–2016, Zika virus (ZIKV) caused major epidemics in French Polynesia, Latin America and the Caribbean, and mosquito-transmitted Zika has been reported in 87 countries and territories (WHO, 2019a). Congenital defects, viral persistence, and maternal-fetal and sexual transmission contributed to Zika becoming a significant global health concern (Mladinich et al., 2017; Pierson and Diamond, 2020). The pathogenesis of dengue in particular is attributed to the existence of 4 different serotypes (DENV1–4) and the fact that although immune responses after primary infection protect against disease with the same serotype, subsequent heterologous infection with a different DENV serotype can increase risk of severe disease (Katzelnick et al., 2017). The latter is thought to be mediated by antibody-dependent enhancement and/or serotype cross-reactive T cells that trigger an exaggerated, skewed immune response to a previously infecting serotype, resulting in a “cytokine storm” that contributes to endothelial permeability and vascular leak (Cardozo et al., 2017; Katzelnick et al., 2017; Rothman, 2011). Plasma leakage is a pathological hallmark of severe dengue, where dysfunction of endothelial microvasculature leads to extravasation of plasma proteins and fluid from the circulatory system into the interstitial compartment, resulting in depleted intravascular volume that may lead to hypovolemic shock and death (Srikiatkhachorn, 2009; Wang et al., 2020).

DENV and ZIKV non-structural protein 1 (NS1) participate in the viral RNA replication complex and are also secreted as glycosylated hexamers in the viremic phase of infection. DENV NS1 plays multiple roles in immunogenicity, host immune evasion, and viral pathogenesis (Glasner et al., 2018; Muller and Young, 2013). Flavivirus NS1 can trigger endothelial hyperpermeability and vascular leak directly in a tissue-specific manner (Puerta-Guardo et al., 2016, 2019), and anti-DENV NS1 and anti-ZIKV NS1 antibodies are protective against lethal disease in mouse models (Beatty et al., 2015; Biering et al., 2021; Wan et al., 2017). Furthermore, ZIKV NS1 increased shedding of endothelial glycocalyx layer (EGL) components and permeability in human trophoblast cell lines and chorionic villi isolated from human placentas (Puerta-Guardo et al., 2020). NS1 also triggers release of vasoactive cytokines from peripheral blood mononuclear cells (PBMCs), contributing to the proinflammatory response induced by virus infection. Notably, higher levels of NS1 in serum/plasma during acute dengue are associated with increased disease severity (Avirutnan et al., 2006; Libraty et al., 2002). Although levels of ZIKV NS1 are lower than DENV NS1 in acute-phase infected individuals (Bosch et al., 2017), its structural similarity to DENV NS1 (Hilgenfeld, 2016) and shared pathways of endothelial barrier dysfunction implicate similar roles in pathogenicity (Puerta-Guardo et al., 2019). These findings highlight the important role of NS1 in dengue and Zika pathogenesis, making this viral protein a promising therapeutic target.

There is no specific treatment for dengue or Zika, and the only currently licensed dengue vaccine (Sanofi-Pasteur) is restricted to previously DENV-immune recipients for safety reasons (Sridhar et al., 2018; WHO, 2019b). Therefore, the development of new therapeutic and/or preventive drugs is urgently needed (Low et al., 2017). Sulfated glycans are a structurally diverse class of compounds for which physicochemical and biological properties depend on sugar composition, molecular weight, chain conformation, degree of substitution, and position of sulfated groups (Yang and Zhang, 2009). The anionic characteristic of sulfo groups on these glycans enables electrostatic interactions with positively-charged glycoproteins on virions, cell membranes, and/or secreted viral glycoproteins such as NS1, potentially inhibiting viral entry into host cells as well as pathogenesis (Ghosh et al., 2009; Kim et al., 2017; Modhiran et al., 2019; Pomin, 2017). Antiviral activity of glycans can also occur via indirect stimulation of immune responses or anti-inflammatory activity (Hayashi et al., 2008; Jiao et al., 2011; Xu et al., 2017).

*Agaricus subrufescens* is a fungus native to Brazil that is widely used and studied due its therapeutic properties, including anti-tumoral, anti-genotoxic, and immunomodulatory activities, mostly related to its polysaccharides (Wisitrassameewong et al., 2012). Here, the (1–6, 1–3)-β-D-glucan isolated from fruiting bodies (FR) of *A. subrufescens* was sulfated to produce the derivative FR-S, which we found to have anti-ZIKV and anti-DENV activity *in vitro* and *in vivo* as well as an inhibitory effect against DENV NS1- and ZIKV NS1-induced endothelial dysfunction *in vitro* and DENV NS1-induced dermal hyperpermeability *in vivo*. Based on these findings, we hypothesize that sulfated glycans could be developed as anti-flavivirus agents targeting both viral replication and pathology of DENV and ZIKV infections.

## 2. Materials and methods

### 2.1. Reagents

The fruiting bodies of *A. subrufescens* Peck (syn *A. brasiliensis* Wasser, previously named *A. blazei* Murrill) were collected in Florianópolis, Santa Catarina state, Brazil. A voucher specimen was deposited in the FLOR Herbarium at the Universidade Federal de Santa Catarina (#FLOR 11797, http://flor.jbrj.gov.br). *A. subrufescens* β-glucan from fruiting bodies (FR) was isolated and chemically characterized as previously described (Cardozo et al., 2013). Briefly, polysaccharides were extracted from dried fruiting bodies with hot water, ethanol precipitated, and purified by filtration, producing a (1 → 6)-(1 → 3)-β-d-glucan with molecular weight (MW) 609 kDa. FR was chemically sulfated using the chlorosulfonic acid/pyridine method, generating a sulfated derivative fully sulfated at C-4 and C-6 terminals and partially sulfated at C-6 of the (1 → 3)-β-d-glucan moiety, which was designated FR-S (MW = 127 kDa). For experiments, freeze-dried FR and FR-S were solubilized in phosphate-buffered saline (PBS), filter-sterilized, and stored at −80°C until use. Recombinant NS1 proteins from ZIKV (Suriname strain Z1106033) and DENV-2 (Thailand strain 16681) were obtained from Native Antigen (United Kingdom) and certified to be endotoxin-free and > 95% purity.

### 2.2. Cell lines and viruses

Human monocytic U937 cells expressing DC-SIGN (kindly provided by Aravinda de Silva, University of North Carolina (UNC), Chapel Hill) were used for antiviral assays. Human pulmonary microvascular endothelial cells (HPMEC, clone ST1–6R) were a gift from J.C. Kirkpatrick, Johannes-Gutenberg University. Human brain microvascular endothelial cells (HBMECs) were a gift from Ana Rodriguez (New York University). DENV-2 (strain 172–06) and ZIKV (strain 2–16) were isolated from serum of infected patients by the National Virology Laboratory, Ministry of Health, Managua, Nicaragua (Montoya et al., 2018). Viruses were propagated in *Aedes albopictus* C6/36 cells (gift from Ralph Baric, UNC Chapel Hill) as previously described (Diamond et al., 2000). Viral titers were determined by focus-forming assay in Vero cells (monkey kidney epithelial cells; ATCC).

### 2.3. Evaluation of FR and FR-S cytotoxicity and anticoagulant activity

Cytotoxicity was assessed by the MTS cellular viability assay (Abcam, USA). In brief, HPMECs (6 × 10^4^ cells/well), HBMECs (8 × 10^4^ cells/well), or U937-DC-SIGN (5 × 10^4^ cells/well) were seeded in 96-well microplates and incubated for 72 hours (h). Cells were treated with medium alone or with different concentrations of compounds in medium for 24 h. Then 200 μg/100 μL of MTS was added to each well, and plates were further incubated for 4 h. Optical densities were read at 490 nm. The 50% cytotoxic concentration (CC_50_) was defined as the concentration that reduced cell viability by 50% when compared to untreated controls.

The anticoagulant activity of FR and FR-S was assayed using the Activated Partial Thromboplastin Time (APTT XL, Pacific Homeostasis) kit, as previously described (Cardozo et al., 2014). Briefly, 50 μL of each compound at 50 μg/mL was mixed with 100 μL of pooled human plasma, then 100 μL of APTT reagent was added. The mixtures were incubated for 3 min at 37°C, then preheated calcium chloride (100 μL, 0.025 M) was added and clotting time was recorded. PBS was used as a negative control. Heparin (Sigma H3393–50KU), a mixture of polyanion chains with MW 6–30 kDa, was used at 0.01 ng/mL as a positive control.

### 2.4. Evaluation of the in vitro anti-DENV and anti-ZIKV activities of FR and FR-S

To evaluate the compounds’ antiviral activity, U937-DC-SIGN cells were infected by DENV-2 or ZIKV [MOI 1, 5 × 10^4^ focus forming units (FFU)], and two different treatments were tested: 1) Simultaneous: cells were treated or not with different concentrations of the compounds at the same time as infection; 2) Post-infection: cells were treated or not after 1 h of infection. After 24 h, cells from both treatments were stained with Alexa Fluor^®^ 647-conjugated anti-flavivirus nonstructural protein 3 (NS3) mouse monoclonal antibody (mAb) E1D8 (Balsitis et al., 2009). Inhibition of viral replication was determined by comparing the number of infected cells in treated versus untreated wells as measured by flow cytometry using an LSR Fortessa cell analyzer (BD Biosciences) and FlowJo software (TreeStar) version 10.2. Celgosivir was used as a positive control.

### 2.5. Evaluation of inhibition of DENV and ZIKV NS1-induced endothelial hyperpermeability

HPMEC monolayers grown on a 24-well Transwell polycarbonate membrane system (0.4 μM, 6.5 mm insert; Corning) were exposed to 10 μg/mL of DENV-2 NS1 and then treated with different concentrations of FR or FR-S (0.12, 0.06, or 0.03 μg/mL). As positive controls, HPMECs with DENV-2 NS1 were left untreated. As basal controls, HPMECs were left untreated. Trans-Endothelial Electrical Resistance (TEER) was measured using a Volt/Ohm Meter (EVOM2, World Precision Instruments), as described previously (Puerta-Guardo et al., 2016). Relative TEER was calculated as the ratio between resistance values obtained in the treated conditions and basal controls. For the ZIKV NS1 assay, we tested FR and FR-S on HBMEC monolayers treated with 10 μg/mL ZIKV NS1. HPMECs were chosen for evaluation of DENV-2 NS1 due to leakage in the lung during severe dengue (pleural effusion), while HBMECs were tested with ZIKV NS1 due to brain pathology seen in severe ZIKV infections.

### 2.6. Evaluation of effects of FR and FR-S on DENV-2 NS1 binding to endothelial cells

HPMEC monolayers grown on gelatin-coated coverslips were pre-chilled at 4°C for 30 min. Then, different concentrations of FR, FR-S, or heparin (positive control) were added simultaneously with 10 μg/mL of His-tagged DENV-2 NS1. After 1 h at 4°C, monolayers were washed, fixed, and stained with Hoechst and anti-His tag mouse mAb (Alexa Fluor^®^ 647 Conjugate, AD1.1.10, Novus Biologicals). Cells were imaged on a Zeiss LSM 710 AxioObserver fluorescence microscope (CRL-Molecular Imaging Center, UC Berkeley). Quantification of NS1 bound to the cell surface was expressed as mean fluorescence intensity. Cells treated with medium alone or compound alone were used as background controls.

### 2.7. Evaluation of interaction between flavivirus NS1 and FR and FR-S

We utilized an intrinsic fluorescence assay to test the interaction of FR, FR-S, and the control compound heparin with DENV and ZIKV NS1, as previously described (Montes-Grajales et al., 2020). Briefly, intrinsic fluorescence of DENV-2 and ZIKV NS1 (200 ng/mL) alone or in the presence of different concentrations of the polysaccharides was read at the excitation wavelength of 295 nm and emission wavelength of 305 nm.

A mobility shift electrophoresis assay in a 0.8% (w/v) agarose gel was performed to analyze DENV-2 NS1 interactions with glycan compounds, as previously described (Oltrogge et al., 2020). Glycan samples at different concentrations were preincubated with 1.5 μg of DENV-2 NS1 for 30 min at room temperature and then loaded onto the gel with sample buffer (0.1 M Tris base (pH 6.8), 4 mM EDTA, 3.2 M Glycerol, 0.05% (w/v) bromophenol blue). The gels were subjected to electrophoresis at 120 V for 1–1.5 h and stained with GelCode Blue (ThermoFisher).

### 2.8. Evaluation of the therapeutic potential of FR-S in the intradermal murine model of dengue vascular leak

We used 5–8-week-old C57BL/6J mice (The Jackson Laboratory) housed in a temperature-controlled environment on a 12-h light/dark cycle, with food and water provided *ad libitum*. All experimental procedures were pre-approved by the UC Berkeley Animal Care and Use Committee, Protocol #AUP-2014–08-6638–2. Briefly, after isoflurane anesthesia, mice were intradermally injected in four different sites on their shaved dorsal dermis with PBS (50 μl), DENV-2 NS1 (15 μg in 50 μL PBS), FR or FR-S (both 0.38 μg in 50 μL PBS) alone, or a mixture of FR or FR-S + DENV-2 NS1 at the same respective concentrations. Immediately following the intradermal injections, 75 μL of 10 kDa dextran-Alexa Fluor 680 (5 mg/mL; Thermofisher) was delivered by retro-orbital injection and 2 h later, mice were euthanized using isoflurane, and the dorsal dermis was removed and placed in Petri dishes. Tissues were scanned using a LI-COR Odyssey CLx fluorescent detection system at a wavelength of 700 nm. Fluorescence intensity was quantified using ImageJ software.

### 2.9. Evaluation of the therapeutic potential of FR-S in a systemic murine model of dengue vascular leak syndrome

Six-to eight-week-old female C57BL/6 interferon (IFN) α/β receptor knockout (*Ifnar*^−/−^) mice, bred in the UC Berkeley Animal Facility, were infected intravenously with 5 × 10^5^ PFU/mouse of DENV-2 D220 (Orozco et al., 2012). Starting on the day of infection through day 5, each mouse was administered 14 mg/kg of FR-S or PBS as vehicle control twice per day via oral gavage. Signs of morbidity were scored as follows: 1- healthy; 2- mild signs of lethargy with some fur ruffling; 3- ruffled fur, hunched, and showing mild signs of lethargy; 4- ruffled fur, hunched, lethargic, and limited mobility; 5- moribund or dead. Once mice reached a score of 4, they were checked twice daily. Mice were euthanized if they achieved a score of 5.

### 2.10. Statistical analysis

Statistical comparisons of test groups for *in vitro* and *in vivo* assays were analyzed by ANOVA followed by multiple comparison tests, as appropriate and as indicated. The 50% effective and cytotoxic concentrations (EC_50_ and CC_50_) were calculated using concentration vs. normalized response curves. The selectivity indices (SI) were calculated as the ratio between cytotoxicity (CC_50_) and antiviral activity (EC_50_). Survival curves were compared by Log-rank (Mantel-Cox) test. Differences were considered statistically significant when p was < 0.05. All analyses were performed in GraphPad Prism Software, version 9.

## 3. Results

### 3.1. FR and FR-S display no significant cytotoxicity or anticoagulant activity

First, we determined cytotoxicity on the studied cells using the MTS assay. FR and FR-S at the maximum concentration tested, 500 μg/mL for FR (0.82 μM) and FR-S (3.94 μM), showed no cytotoxicity on HPMECs, HBMECs, or U937-DC-SIGN cells (Table 1). A precaution in the development of sulfated saccharides as drug candidates is their potential anticoagulant activity, since anticoagulant effects may enhance pathogenesis, especially for DENV infection where bleeding is a frequent warning sign. *In vitro* anticoagulant activity was measured using normal human plasma in the APTT assay. Coagulation time values obtained for plasma mixed with PBS alone, 50 μg/mL FR, or 50 μg/mL FR-S were 35.75 ± 0.48, 36.40 ± 1.31, and 40.67 ± 2.05 s, respectively. In contrast, no coagulation occurred with heparin at 0.01 ng/mL. Thus, FR and FR-S had no significant anticoagulant activity even when tested at a concentration 5 × 10^5^ times higher than heparin (0.01 ng/mL). These results indicated that FR and FR-S were safe for further testing.

### 3.2. FR-S inhibits DENV and ZIKV replication when added simultaneously with viral infection

**Fig. 1.**
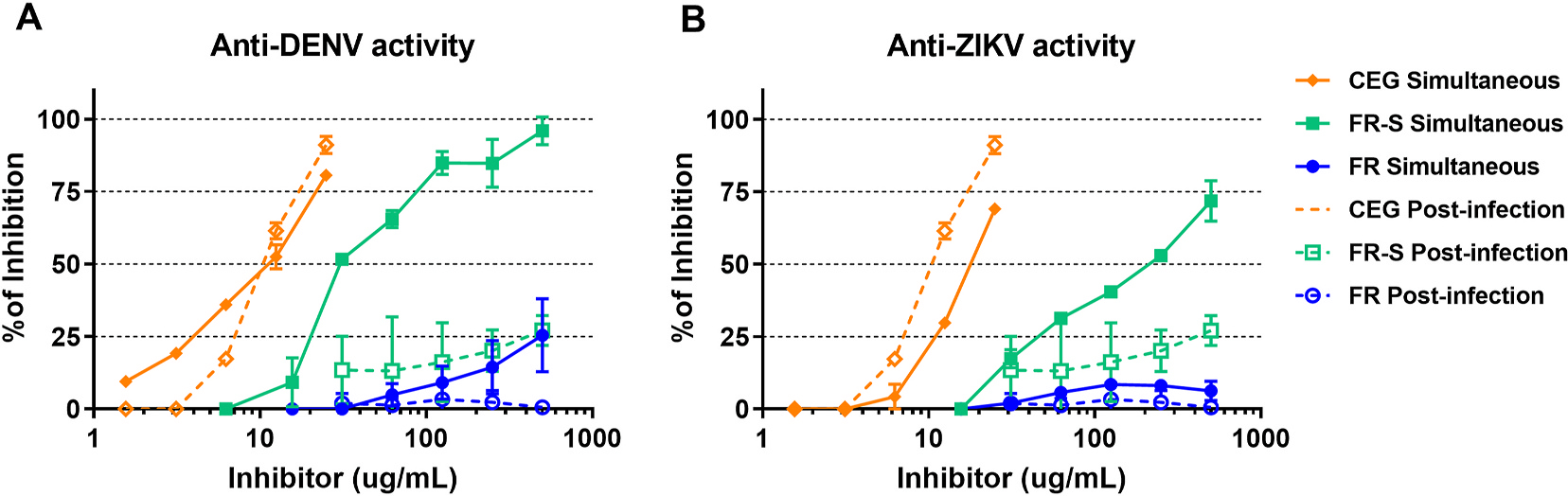
FR-S, but not FR, inhibits DENV-2 and ZIKV replication. U937-DC-SIGN cells were infected with (A) DENV-2 (Nicaraguan strain 172–06) or (B) ZIKV (Nicaraguan strain 2–16) and treated simultaneously or 1 h post-infection with different concentrations of FR, FR-S, or the positive control celgosivir (CEG). Viral replication was quantified by flow cytometry with intracellular labeling of nonstructural protein 3 (NS3), indicating active replication. Mouse anti-NS3 Alexa Fluor 647 conjugated mAb (E1D8) was used for NS3 staining.

We then determined the antiviral efficacy of FR and FR-S *in vitro* by detecting the presence of NS3, which indicates active viral replication in infected U937-DC-SIGN cells. Cells were infected with DENV-2 or ZIKV and treated simultaneously or 1 h post-infection with different concentrations of the compounds. The number of cells with active replicating viruses was quantified by flow cytometry. We found that FR-S significantly inhibited replication of both DENV-2 and ZIKV when added simultaneously with viral infection (Fig. 1, Table 1). FR-S inhibition of DENV-2 replication was greater than its effect on ZIKV replication. No inhibitory effect was observed when compounds were added 1 h post-infection, at the maximal concentration tested (500 μg/mL). We also performed a Focus Reduction Assay in Vero cells infected with DENV-2 or ZIKV, and the results showed that neither FR nor FR-S exhibited anti-DENV or anti-ZIKV activity with pre-treatment of cells or treatment 1 h post-infection at 500 μg/mL (data not shown). Together, these results indicate that inhibition of viral entry by targeting the virus is the likely mechanism of action of FR-S. FR only displayed slight anti-DENV activity (25.5% of inhibition at 500 μg/mL) in the simultaneous treatment condition, indicating that sulfation is essential for the antiviral effect. Celgosivir, an alpha-glucosidase I inhibitor used as positive control, presented anti-DENV and anti-ZIKV activity in both simultaneous and post-infection treatments.

### 3.3. FR and FR-S reduce DENV and ZIKV NS1-induced endothelial hyperpermeability

**Fig. 2.**
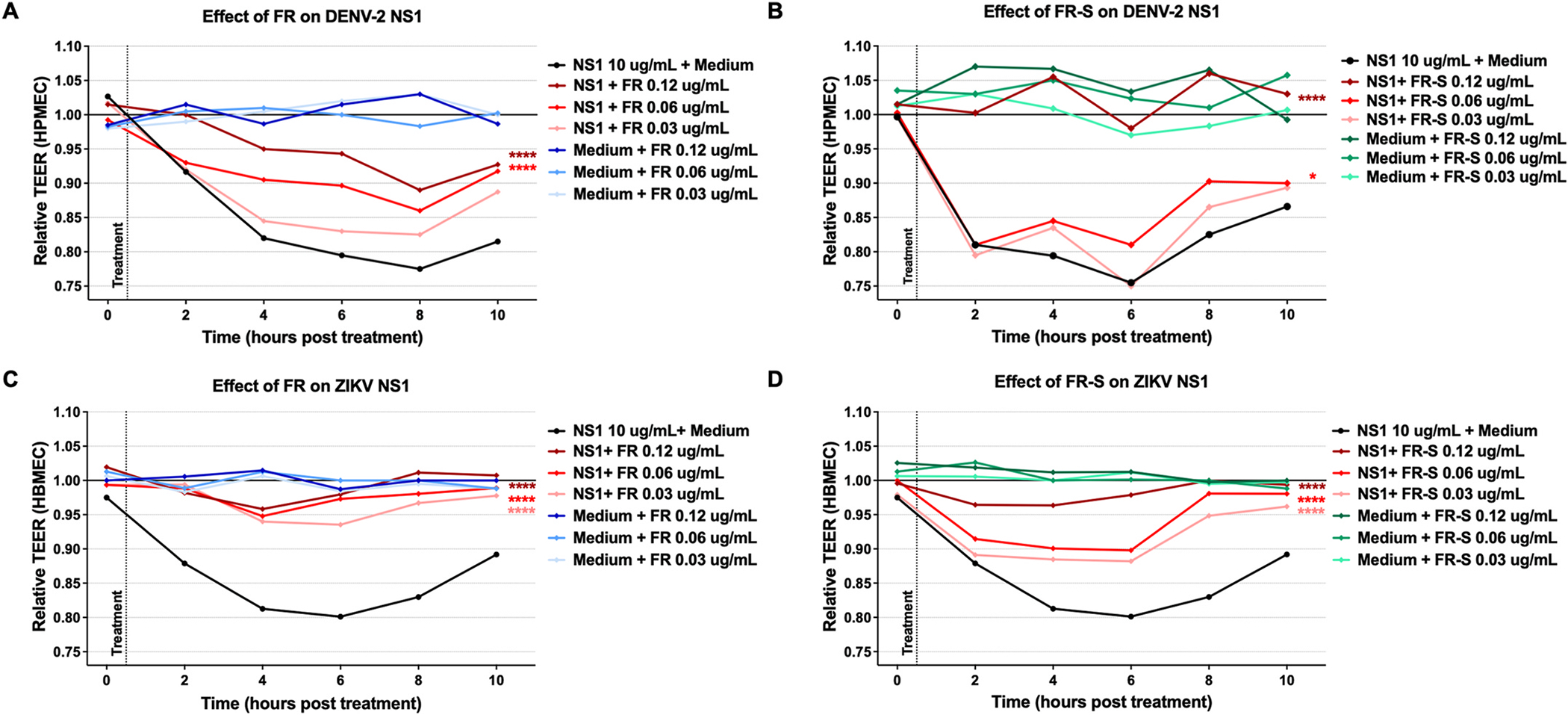
FR and FR-S reduce DENV and ZIKV NS1-induced endothelial dysfunction. (A, B) HPMEC monolayers were incubated with or without 10 μg/mL of DENV-2 NS1 and then treated with FR (A) or FR-S (B) at three different concentrations, as indicated. (C, D) HBMEC monolayers were incubated with or without 10 μg/mL of ZIKV NS1 and then treated with FR (C) or FR-S (D) at three different concentrations, as indicated. TEER was measured 2–10 h post-treatment. The area under the curve (AUC) of each treatment was compared to treatment with NS1 alone via one-way ANOVA + Dunnett’s multiple comparisons test. Asterisks indicate statistically significant differences, with *, *p* < 0.05; **, *p* < 0.005; ***, *p* < 0.001; or ****, *p* < 0.0001.

TEER is a proxy for barrier permeability *in vitro*, and it can be used to test the efficacy of compounds that protect against endothelial barrier dysfunction. Therefore, we used TEER to measure the effect of FR and FR-S on DENV and ZIKV NS1-induced endothelial disruption. FR significantly inhibited TEER reduction induced by DENV-2 NS1 treatment of HPMECs at 0.12 and 0.06 μg/mL, with > 50% inhibition. FR-S completely inhibited DENV-2 NS1-mediated endothelial hyperpermeability at 0.12 μg/mL. Similarly, 0.12 μg/mL of FR or FR-S significantly inhibited (> 80%) ZIKV NS1-induced disruption of HBMECs (Fig. 2). These data demonstrate the potential of FR and FR-S to prevent flavivirus NS1-induced hyperpermeability.

### 3.4. FR and FR-S interfere with DENV NS1 binding to HPMECs

**Fig. 3.**
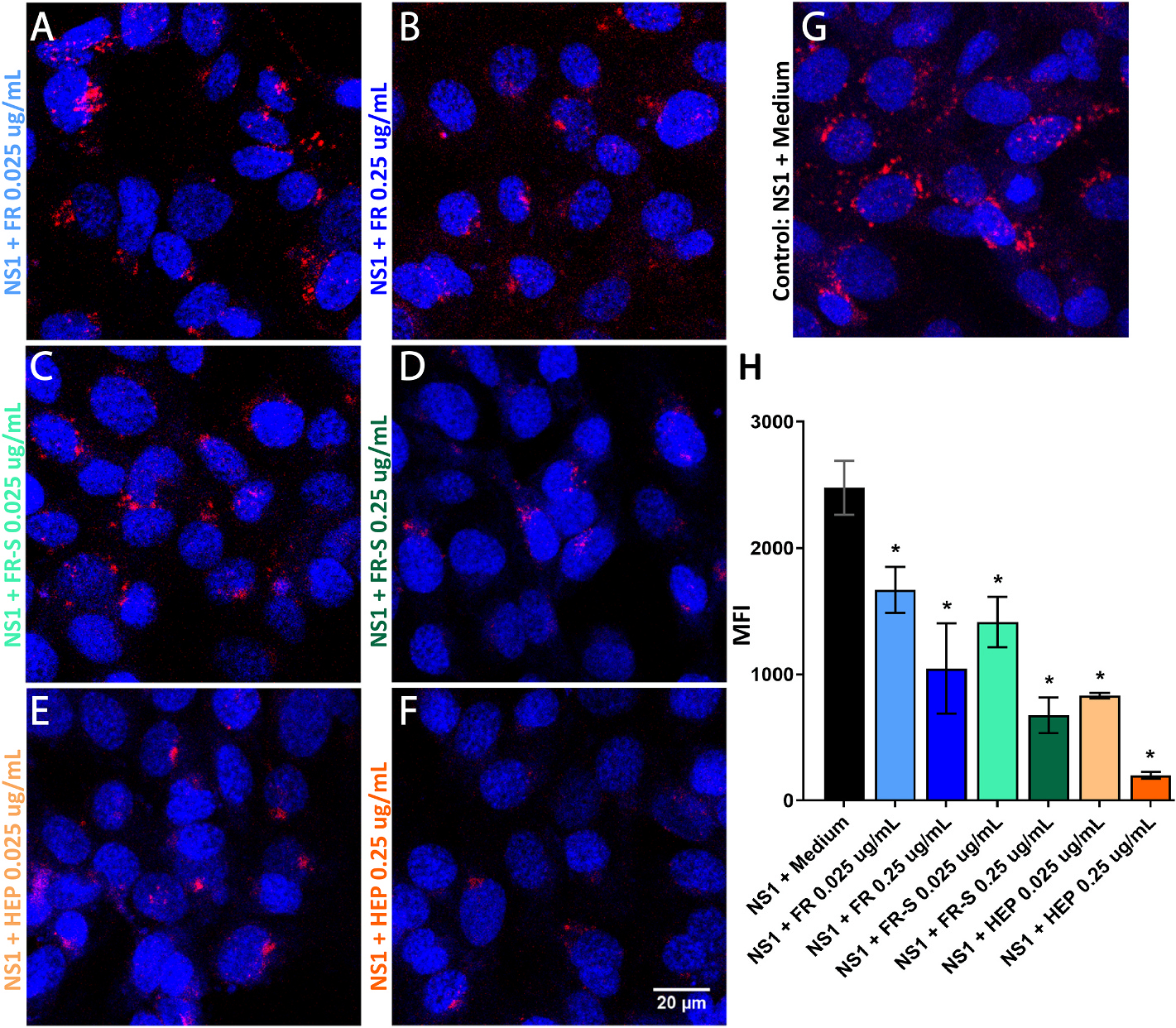
FR and FR-S block DENV-2 NS1 binding to HPMECs. HPMEC monolayers were incubated with 10 μg/mL of His-tagged DENV-2 NS1 and treated with FR **(A, B)**, FR-S **(C, D)**, or heparin **(E, F)** at two different concentrations (0.25 and 0.025 μg/mL) for 1 h. Afterwards, cells were fixed and stained with Hoechst (blue) and anti-6x-His-tag mAb conjugated to Alexa 647 (red). Treatments were compared to NS1 + Medium group **(G)** by one-way ANOVA + Dunnett’s multiple comparison test. Asterisks (*) indicate significant differences, with *p* < 0.05. Quantification of NS1 protein bound to the cells surface was expressed as mean fluorescence intensity (MFI) as shown in panel **H**.

We next investigated the mechanism of action by which FR/FR-S inhibit NS1-mediated endothelial hyperpermeability. Given that flavivirus NS1 attaches to cell surface glycosaminoglycans (GAGs), such as heparan sulfate (HS) and chondroitin sulfate (CS), sulfated polysaccharides are strong candidates as NS1 binding inhibitors (Avirutnan et al., 2007; Wang et al., 2019). Therefore, we tested if FR and FR-S prevent NS1 binding to the target cells. Using confocal microscopy, we found that treatment with 0.25 μg/mL of FR inhibited 64% of DENV-2 NS1 binding to HPMECs, whereas treatment with 0.25 μg/mL of FR-S inhibited 73% of DENV-2 NS1 binding (Fig. 3). Heparin, a non-branched sulfated polysaccharide clinically used as an anticoagulant, also inhibited NS1 binding. These findings suggest that one mechanism by which FR and FR-S prevent NS1-mediated endothelial barrier dysfunction involves inhibition of NS1 binding to endothelial cells. The higher level of inhibition by FR-S implies that sulfation increases the inhibition of NS1 binding to endothelial cells, which is consistent with previous literature (Avirutnan et al., 2007; Modhiran et al., 2019).

### 3.5. FR and FR-S interact with flavivirus NS1

**Fig. 4.**
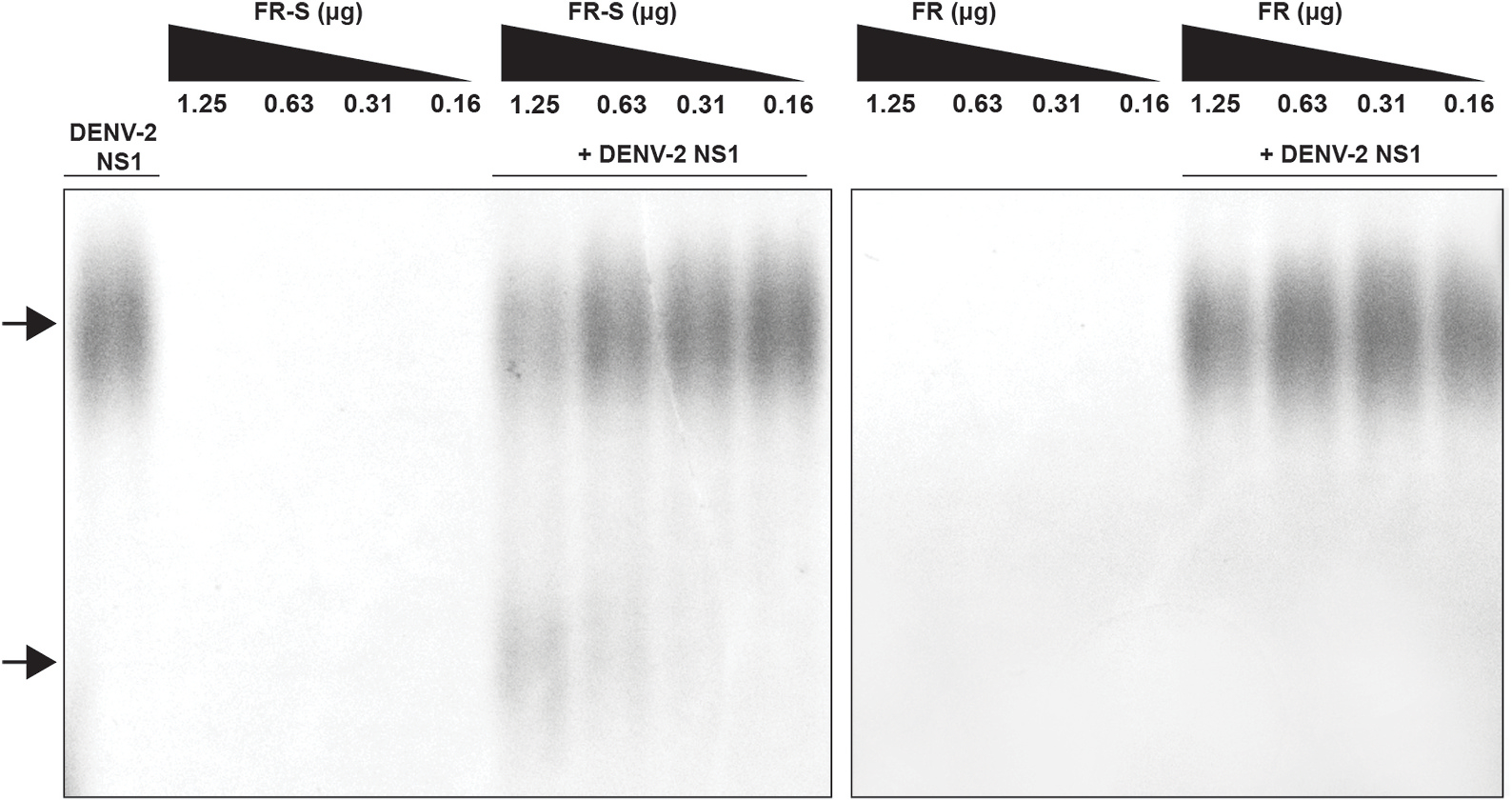
FR-S interacts with DENV-2 NS1. An agarose gel electrophoresis was performed with FR or FR-S at four different concentrations (1.25–0.16 μg) in the presence or not of 1.5 μg of DENV-2 NS1. FR-S at 1.25 and 0.63 μg shifted the mobility of NS1 (bottom arrow) as compared to NS1 alone (top arrow).

Results of an intrinsic fluorescence assay showed decreased fluorescence emission of NS1 in the presence of the tested glycan compounds. Results presented in Supplementary Fig. S1 indicate an interaction of FR-S with DENV NS1, as well as between FR and FR-S and ZIKV NS1. Results of an electrophoretic mobility assay showed that FR-S shifted the mobility of DENV-2 NS1 in agarose gel electrophoresis in a concentration-dependent manner, further indicating an interaction between FR-S and DENV-2 NS1 (Fig. 4).

### 3.6. FR-S inhibits DENV NS1-induced vascular leak in the mouse dermis

**Fig. 5.**
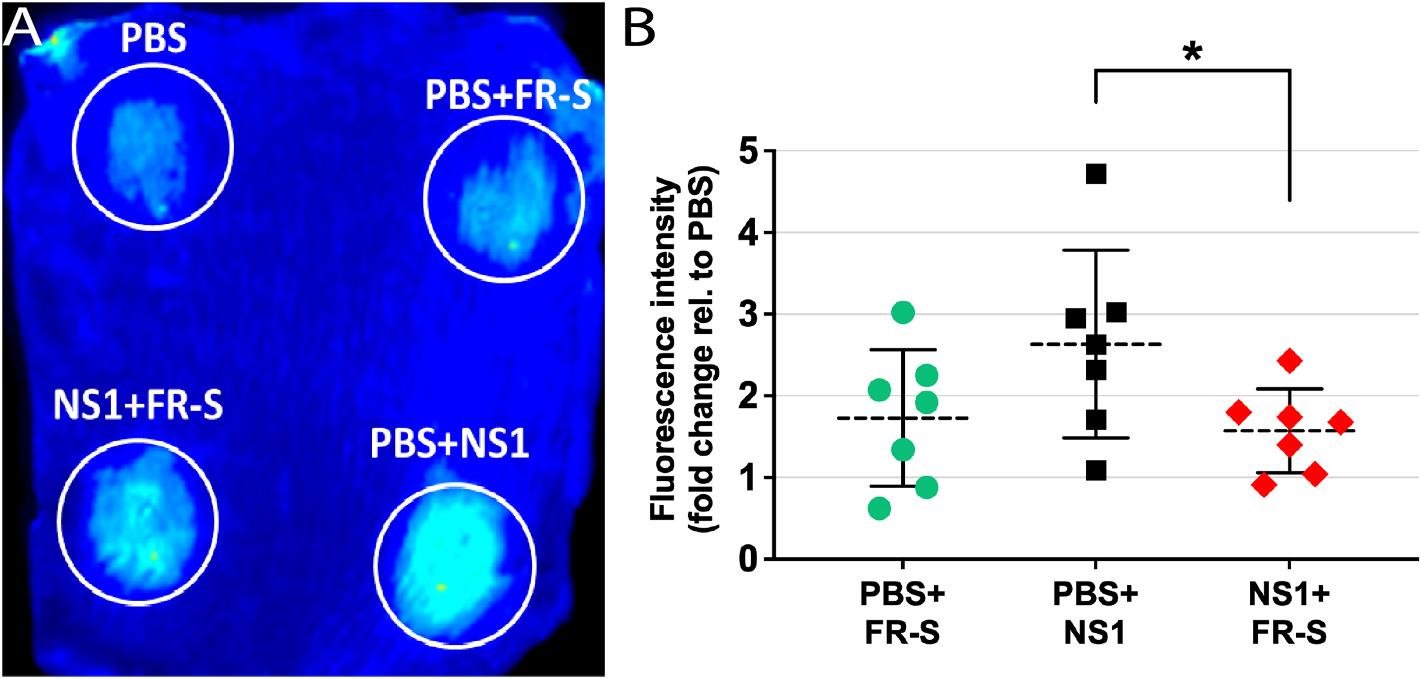
FR-S inhibits DENV-2 NS1-induced vascular leak in the mouse dermis. (A) Each C57BL/6 mouse was intradermally injected in the dorsal dermis with four different treatments (PBS, PBS + FR-S, PBS + NS1, and NS1 + FR-S) and then administered Alexa Fluor 680-conjugated 10 kDa dextran (5 mg/mL) via retro-orbital injection. Two hours later, mice were euthanized, and the dorsal dermis was removed and scanned using a LI-COR Odyssey CLx Imaging System. **(B)** Images were analyzed using ImageJ to obtain mean fluorescence intensity values, which were expressed as fold-change in comparison to PBS treatment. Treatment groups (N = 6) were compared by one-way ANOVA + Tukey’s test. Asterisk (*) indicates significant difference, with *p* < 0.05.

Given their promising *in vitro* activity on DENV and ZIKV NS1-induced endothelial disruption, we assessed the ability of FR and FR-S to inhibit dermal endothelial permeability using a fluorescent dextran-adapted local vascular leak assay in C57BL/6 mice. While FR had no significant effect (data not shown), FR-S significantly reduced (41.8%, *p* = 0.0476) vascular leak induced by DENV-2 NS1 injected intradermally (Fig. 5). These data demonstrate the protective effects of FR-S on NS1-induced leakage *in vivo*.

### 3.7. FR-S reduces morbidity and mortality of mice infected with DENV-2

**Fig. 6.**
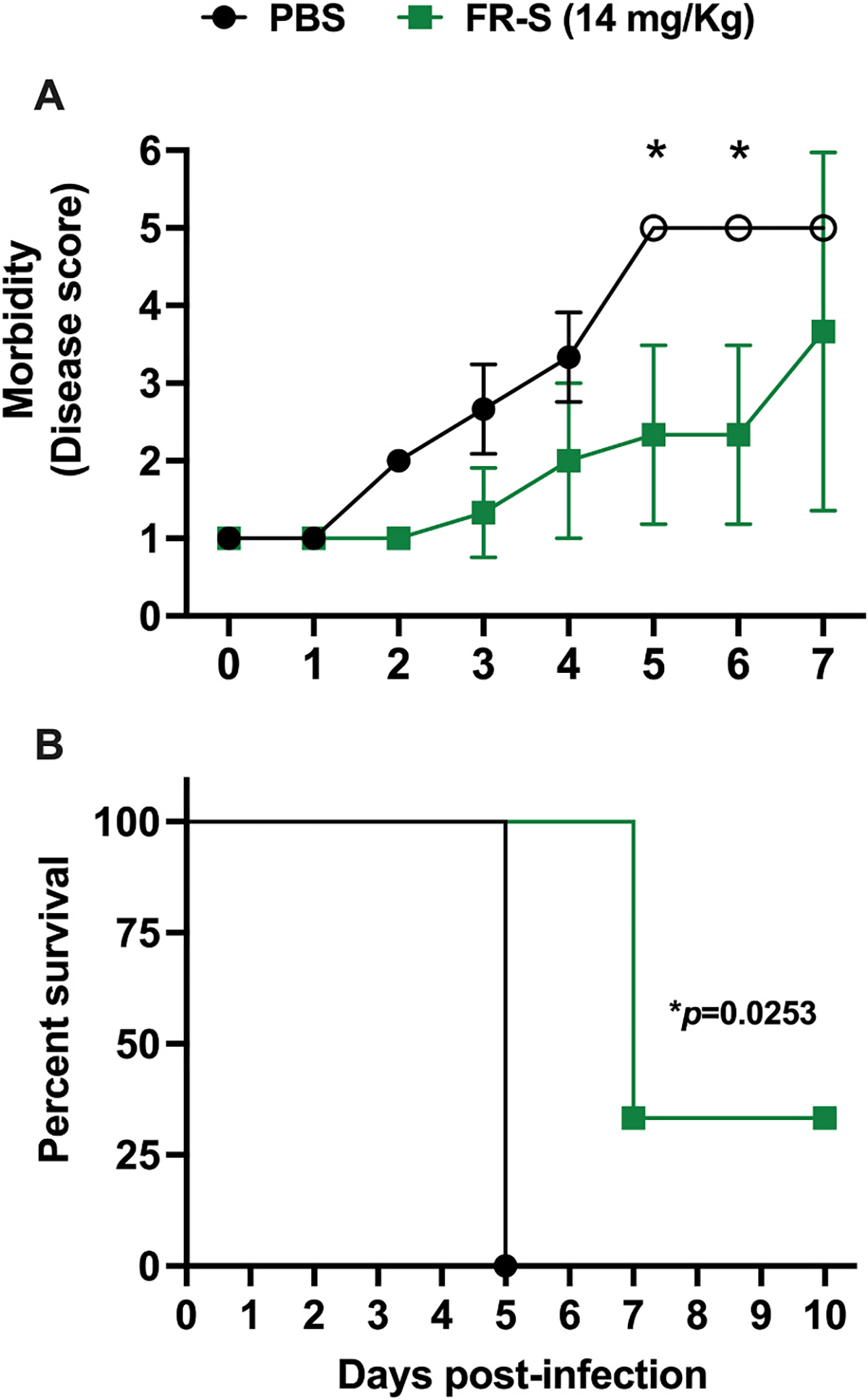
FR-S inhibits morbidity and mortality in a mouse model of dengue vascular leak syndrome. (A) C57BL/6 *Ifnar*^−/−^ mice infected i.v. with a lethal dose of the DENV-2 strain D220 (5 × 10^5^ PFU) were treated with a non-toxic dose of FR-S (14 mg/kg, n = 3) or PBS (n = 3) via oral gavage twice per day for 5 days, starting on the day of infection. Mice were monitored daily and scored according to a scale of 1–5 described in Methods, where 1 represents healthy mice and 5 represents mice that succumbed to infection. Mean disease scores were compared separately each day by one-way ANOVA + Šídák’s multiple comparisons test. **(B)** A Kaplan–Meier survival curve of mice up to 10 days post-infection compared using a Mantel-Cox log-rank test. (*) indicates significant difference, with *p* < 0.05.

In order to assess *in vivo* efficacy of FR-S, C57BL/6 *Ifnar*^−/−^ mice infected with a lethal dose of the DENV-2 strain D220 in our dengue model of vascular leak syndrome (Orozco et al., 2012) were treated with a non-toxic dose of FR-S (14 mg/kg) twice per day for 5 days starting on the day of infection. Results show that FR-S oral treatment reduced morbidity of mice when compared with vehicle control group, with significant differences on days 5 and 6 post-infection. Mice treated with FR-S also showed significant reduction in mortality caused by DENV infection (Fig. 6).

## 4. Discussion

Given the unique chemical configuration of the fungal cell wall glycan isolated from *A. subrufescens* fruiting bodies (FR) and its sulfated derivative FR-S, in addition to its previously reported antiviral properties, we tested their capacity to prevent DENV and ZIKV replication as well as DENV-2 NS1- and ZIKV NS1-induced endothelial dysfunction. We found that FR-S, but not FR, significantly inhibited DENV-2 and ZIKV replication in human U937-DC-SIGN cells when added simultaneously with viral infection. Furthermore, FR and FR-S inhibited endothelial barrier dysfunction induced by DENV-2 NS1 and ZIKV NS1 and both inhibited DENV-2 NS1 binding to the ECs. However, only FR-S significantly inhibited DENV-2 NS1-induced vascular leak in the mouse dermis. Moreover, FR-S inhibited morbidity and mortality of mice infected with a lethal dose of DENV-2 in a mouse model of dengue vascular leak syndrome. The inhibition by FR and FR-S of NS1 binding to ECs, together with the known interaction between flavivirus NS1 and glycosaminoglycans, especially with sulfated moieties, make it likely that these glycans bind NS1, preventing its interaction with host cells and downstream pathology. In fact, intrinsic fluorescence and agarose mobility shift assays indicated interaction of FR-S with DENV-2 and ZIKV NS1 and of FR with ZIKV NS1.

The development of antivirals for dengue is especially challenging due to the complex, incompletely understood pathogenesis and the co-circulation of four distinct serotypes (Lai et al., 2017; Nedjadi et al., 2015; Noble and Shi, 2013). In the case of Zika, an effective treatment that is neuroprotective and safe for pregnant women is also needed (Saiz and Martín-Acebes, 2017). At least four strategies can be envisioned for the treatment of dengue and Zika: inhibition of viral infection, host factors essential for viral replication, and/or pathophysiological pathways of disease, as well as activation of antiviral immune responses (Noble and Shi, 2013). Here, we show the dual effect of FR-S inhibiting both viral infection and NS1-mediated pathophysiology.

The interaction between the envelope protein (E) and GAGs is critical for attachment of flaviviruses to cells (e.g., dengue, Zika, and yellow fever virus) (Kim et al., 2017). Sulfated glycans can mimic GAGs, coating virus particles and potentially preventing virus attachment and entry into cells. In fact, other sulfated polysaccharides have been shown to have anti-DENV activity by blocking virus attachment (Hidari et al., 2008; Lee et al., 2006; Muschin et al., 2016). FR-S displayed *in vitro* anti-HSV-1 and anti-HSV-2 activities in both simultaneous and post-infection treatments (Cardozo et al., 2014). Beta-glucan fractions from *Lentinus edodes*, *Boletus edulis* and *Pleurotus ostreatus* mushrooms also displayed anti-HSV-1 activity *in vitro* (Santoyo et al., 2012). A β-glucan blend from mycelia of Shiitake, Reishi, Agaricus, and Chaga mushrooms displayed antiviral activity by enhancing immune responses against influenza in mice (Vetvicka and Vetvickova, 2015). Thus, sulfated β-glycans are a promising class of broad-spectrum antivirals acting mainly as viral entry inhibitors and stimulators of antiviral immune responses.

Flavivirus NS1 plays a role in pathogenesis by directly disrupting the endothelial glycocalyx and tight and adherens junctions, as well as triggering release of vasoactive cytokines from PBMCs, all leading to endothelial barrier dysfunction (Beatty et al., 2015; Glasner et al., 2018; Modhiran et al., 2015; Puerta-Guardo et al., 2016, 2020). We have shown that an anti-NS1 mAb, 2B7, is protective against flavivirus NS1-induced endothelial dysfunction as well as lethal DENV infection and DENV-induced vascular leak in mice (Biering et al., 2021). In addition, anti-ZIKV NS1 mAbs have been shown to protect against ZIKV infection in both non-pregnant and pregnant mice (Bailey et al., 2018; Wessel et al., 2020). Thus, the contribution of NS1 to flavivirus pathogenesis makes this viral toxin an attractive therapeutic target. In addition to the virion, DENV and ZIKV NS1 proteins secreted from infected cells attach to cell surfaces via GAGs, including HS and/or CS (Avirutnan et al., 2007; Glasner et al., 2018). Therefore, blocking NS1 interaction with target cells (endothelial and monocytic cells) using exogenous sulfated glycans, such as FR-S, could represent a promising antiviral strategy, namely, preventing disease. In fact, a recent study showed that a HS mimic oligosaccharide had anti-DENV and anti-DENV NS1 activity *in vitro* and in mouse models of vascular leakage (Modhiran et al., 2019). In sum, dual targeting of flavivirus infection and NS1 effects by sulfated glycans is an approach not yet sufficiently explored and may represent an attractive option for flavivirus therapeutics.

## 5. Conclusions

The sulfated derivative of *A. subrufescens* β-glucan (FR-S) displayed efficacy against DENV-2 and ZIKV infection as well as DENV- and ZIKV-NS1-induced endothelial disruption *in vitro. In vivo*, FR-S reduced DENV-2 NS1-induced dermal vascular leak as well as morbidity and mortality in a systemic model of lethal DENV-2 vascular leak syndrome. Compound sulfation was crucial for effective antiviral and anti-DENV-2 NS1 activity. These findings stimulate further exploration of FR-S and other sulfated glycans as candidates for dengue treatment alone or as combination therapies with compounds with different mechanisms of action.

## Supplementary Material

1

## Figures and Tables

**Table 1:** Cytotoxicity and antiviral activity of FR and FR-S.

		EC_50_ (SI)			
		
		Simultaneous treatment		Post-infection treatment	
		
Sample	CC_50_	DENV-2 (Strain 172–06)	ZIKV (Strain 2–16)	DENV-2 (Strain 172–06)	ZIKV (Strain 2–16)

FR	>500	NI	NI	NI	NI
FR-S	>500	36.50 ± 2.55 (>13.70)	188.70 ± 12.87 (>2.78)	NI	NI
CEG	>100	10.01 ± 0.53 (>9.99)	17.99 ± 0.13 (>5.56)	4.93 ± 0.11 (>20.28)	10.75 ± 0.52 (>9.30)

CC_50_: 50% cytotoxic concentration for U937-DC-SIGN cells (μg/mL); EC_50_: 50% effective concentration (μg/mL); Selectivity index values (SI = CC_50_/EC_50_) are presented between parenthesis; NI: No inhibitory activity; FR: *A. subrufescens* polysaccharide; FR-S: Sulfated *A. subrufescens* polysaccharide; CEG: Celgosivir. Values represent the mean ± SD of three independent experiments.

## Highlights:

Sulfated β-glucans inhibit DENV and ZIKV infection, morbidity, and mortality, as well as NS1-mediated endothelial disruption and vascular leak.
