# Supplementary material for: Sulfated β-glucan from Agaricus subrufescens inhibits flavivirus infection and nonstructural protein 1-mediated pathogenesis
Source: Antiviral Res. Author manuscript; Available in PMC 2023 Aug 11. (PMC10416543; doi:10.1016/j.antiviral.2022.105330)
Supplement: 1 [file NIHMS1915949-supplement-1.docx]

**Supplementary figure 1**


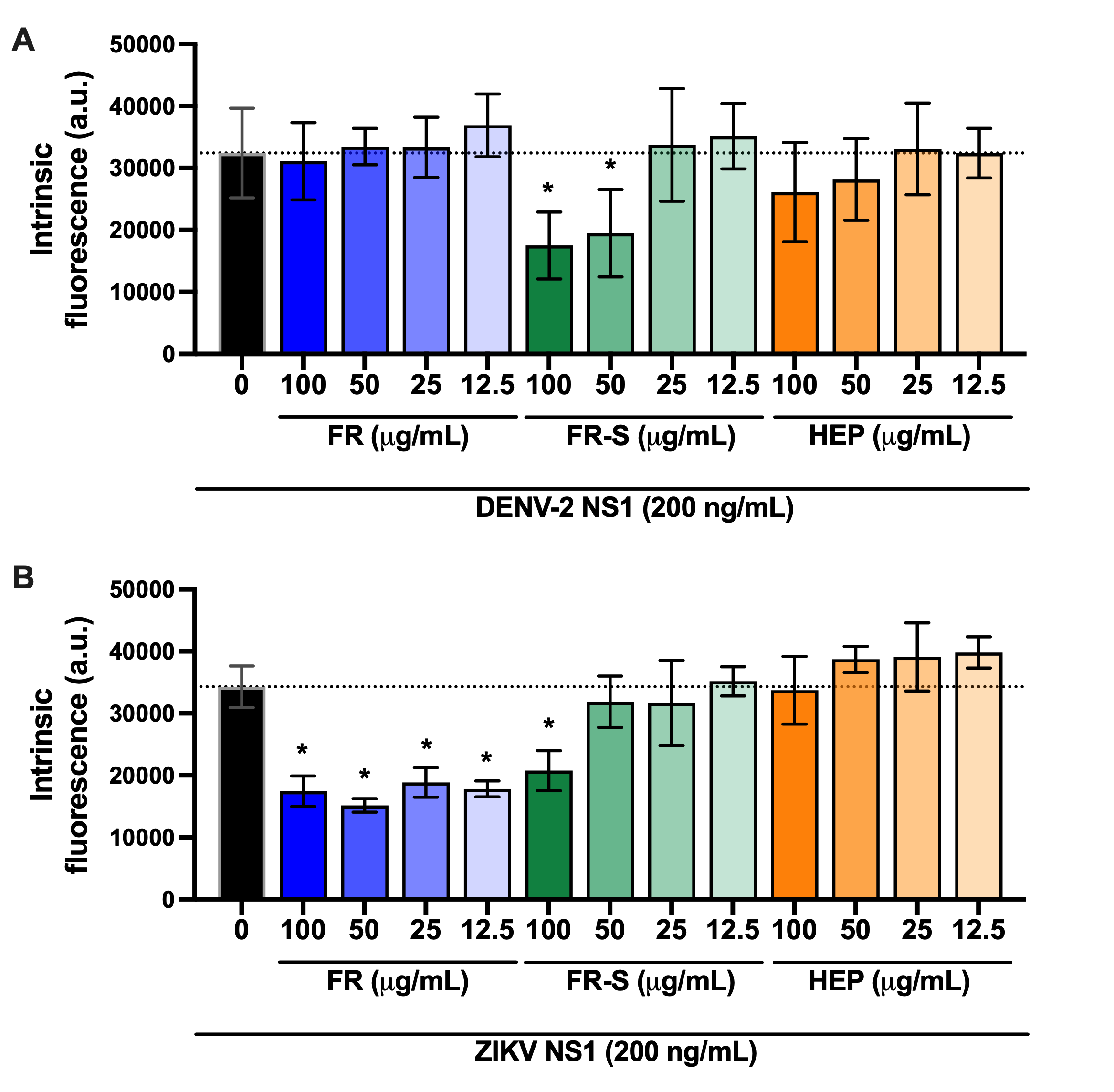


Supplementary figure 1. FR and FR-S interact with flavivirus NS1. An intrinsic fluorescence assay was performed using 200 ng/mL of DENV-2 NS1 (A) or ZIKV NS1 (B) diluted in PBS in the presence or not of different concentrations (100-12.5 µg/mL) of the *Agaricus subrufescens* beta-glucan (FR), its sulfated derivative (FR-S), or heparin (HEP), using an excitation wavelength of 295 nm and emission wavelength of 305 nm. Fluorescence measurements were acquired using a Spectra Max (M3) microplate reader (Molecular Devices) equipped with a dual monochromator spectrofluorometer system. Fluorescence units obtained from 2 individual experiments including 4 repeats each were plotted using GraphPad software. Mean fluorescence values of compounds + NS1 were compared to NS1 alone by one-way ANOVA + Dunnett's multiple comparison test with *p*<0.05 considered significant (*).
